# Bioluminescent Imaging of *Trypanosoma brucei* Shows Preferential Testis Dissemination Which May Hamper Drug Efficacy in Sleeping Sickness

**DOI:** 10.1371/journal.pntd.0000486

**Published:** 2009-07-21

**Authors:** Filip Claes, Suman K. Vodnala, Nick van Reet, Nathalie Boucher, Hilda Lunden-Miguel, Theo Baltz, Bruno Maria Goddeeris, Philippe Büscher, Martin E. Rottenberg

**Affiliations:** 1 Institute of Tropical Medicine Antwerp, Department of Parasitology, Antwerp, Belgium; 2 Katholieke Universiteit Leuven, Deptartment of Biosystems, Leuven, Belgium; 3 Karolinska Institute, Microbiology and Tumorbiology Center, Stockholm, Sweden; 4 Laboratoire de Microbiologie Cellulaire et Moléculaire et Pathogénicité UMR/CNRS-5234, Université Victor Segalen Bordeaux II, Bordeaux, France; Yale School of Public Health, United States of America

## Abstract

Monitoring *Trypanosoma* spread using real-time imaging *in vivo* provides a fast method to evaluate parasite distribution especially in immunoprivileged locations. Here, we generated monomorphic and pleomorphic recombinant *Trypanosoma brucei* expressing the *Renilla* luciferase. *In vitro* luciferase activity measurements confirmed the uptake of the coelenterazine substrate by live parasites and light emission. We further validated the use of *Renilla* luciferase-tagged trypanosomes for real-time bioluminescent *in vivo* analysis. Interestingly, a preferential testis tropism was observed with both the monomorphic and pleomorphic recombinants. This is of importance when considering trypanocidal drug development, since parasites might be protected from many drugs by the blood-testis barrier. This hypothesis was supported by our final study of the efficacy of treatment with trypanocidal drugs in *T. brucei*-infected mice. We showed that parasites located in the testis, as compared to those located in the abdominal cavity, were not readily cleared by the drugs.

## Author Summary

Human African trypanosomiasis or sleeping sickness, caused by two subspecies of *Trypanosoma brucei*, is endemic in Subsaharan Africa. There is no vaccine and the currently used drugs are toxic and can cause severe side effects and even death. At present, we do not know how and when parasites can leave the blood and penetrate into organs (especially the brain). Such knowledge will be very helpful to develop and validate new drugs that can clear the parasite from both the blood and the tissues. In this study, we developed a novel technique allowing us to track the parasites in a live animal by the use of light signals. By following the luminescent parasites in the mouse we showed that, interestingly, the organisms migrate very early in infection to the testes. Here, they may be protected from the immune system and from drugs. Indeed when treating the mice with a sub-optimal dose of medicine, the parasites in this location were not cleared and subsequently could cause a reinvasion into the blood of the host.

## Introduction

Human and animal African trypanosomoses are important protozoan infections endemic in Africa, Latin America and Asia, caused by several species such as *Trypanosoma brucei*, *T. evansi*, *T. equiperdum*, *T. congolense* and *T. vivax*. Different species, strains within the species and clones within various strains show different tissue tropism that may further vary within hosts [1]. Currently no vaccines against human and animal trypanosomoses are available; and a limited range of drugs exist to treat these diseases. Moreover, most of the drugs used in second stage sleeping sickness show a high toxicity while in animal trypanosomosis drug resistance becomes more and more problematic [2]. Our current knowledge on tissue tropism, mechanisms by which trypanosomes invade and spread into tissues, the temporal course of invasion and the drug accessibility to trypanosomes in tissues, is incomplete. To complement classical anatomopathological examinations, real-time biophotonic imaging seems straight forward. Bioluminescence *in vivo* imaging allows longitudinal monitoring of an infection in the same animal, a desirable alternative to analyzing a number of animals at many time points during the course of the infection. To date, most bioluminescence models have been generated to monitor pathogenic bacterial infections, such as *Salmonella*, and bacterial meningitis [3],[4]. Among pathogenic protozoa only *Plasmodium berghei*, *Leishmania amazonensis* and *Toxoplasma gondii* have been engineered to express the firefly luciferase and used in bioluminescence imaging [5]–[7]. To our knowledge, no bioluminescent model for trypanosomes has been developed. Here we report the generation of recombinant *Renilla luciferase expressing* parasites, and the validation of the use of a real time biophotonic detection of parasites to study the dissemination of African trypanosomes in mice *in vivo* and the efficiency of treatment with trypanocidal drugs *in vitro* and *in vivo*.

## Materials and Methods

### Construction of luminescent *T. brucei*, transfections and cell culture

The Rluc gene was PCR-amplified from pGL4.70 (Promega) and cloned into the pHD309 plasmid [8] using the In-Fusion PCR cloning kit (ClonTech). Plasmids were screened via HindIII/BamHI double restriction-digestion, sequenced, and those with the correct insert in frame were selected and propagated in *E. coli*. Ten µg of the Rluc-pHD309 plasmid was linearized using NotI (10 U, 3 hours at 37 C). *T. brucei* bloodstream forms (Lister 427 host cell line 90-13 and AnTat 1.1^E^) were cultured at 37°C, 5% CO_2_ in IMDM medium (Gibco) supplemented with 10% (v/v) heat-inactivated fetal calf serum, 36 mM sodium bicarbonate, 136 µg.ml^−1^ hypoxanthine, 39 µg.ml^−1^ thymidine, 110 µg.ml^−1^ NaPyruvate, 28 µg.ml^−1^ bathocuproine, 0.25 mM β-mercaptoethanol, 2 mM L-cystein and 62.5 µg.ml^−1^ kanamycin [9]. A pellet of 2×10^7^ parasites was resuspended in 400 µl warm cytomix (2 mM EGTA, 120 mM KCL, 0.15 mM CaCl_2_, 10 mM K_2_HPO_4_/KH_2_PO_4_ pH 7.6, 25 mM Hepes, 0.5%Glucose, 1 mM Hypoxanthine, 100 µg/ml BSA, 5 mM MgCL_2_) [10] and transferred into a 4 mm cuvette; 10 µg of linearized DNA construct was added and left for one minute at 37°C. Subsequently the mixture was pulsed once in a Gene Pulse Xcell square wave electroporator at 1250 V, 25 Ohm, 50 µF and transfected cells were added to 12 ml of preheated IMDM, plated in a 24-well plate (24-time 500 µl) and incubated at 37°C for 24 h. Next, 500 µl of preheated IMDM containing 10 µg/ml hygromycin were added to obtain a final selection concentration of 5 µg/ml. Positive clones were evident at 6 days post transfection.

### Immunofluorescence and microscopy

Transfected *Trypanosoma brucei brucei* AnTat 1.1^E^ bloodstream form trypanosomes were grown for 3 days in mice. Ten microlitres of blood were spread over a microscope slide and fixed with acetone for 15 minutes. The fixed cells were incubated at room temperature with primary and secondary antibodies for 1 h and 30 min, respectively, and washed two times for 5 min with PBS after each of the incubations. The primary antibody, monoclonal mouse anti-*Renilla* luciferase (Millipore), was diluted 1∶2 in PBS. The secondary antibody, fluorescein isothiocyanate (FITC)-conjugated goat anti-mouse (Jackson), was diluted 1∶100 in a solution of 0.1 mg.ml^−1^ Evans Blue and 1 µg.ml^−1^ DAPI in PBS. Cells were analyzed on an Olympus BX-41 UV microscope, and images were captured by a Colorview II camera (Soft Imaging Systems) and Cell_D software (Soft Imaging Systems) was used for analysis.

### *In vitro* bioluminescence measuring

The *Renilla* Luciferase Assay System (Promega) was used to measure *in vitro* luciferase activity. Non-transformed *T. brucei* Lister 427 and *T. brucei* 427–Rluc-pHD309 clones were grown up to a total of 1×10^7^ parasites (10 ml of 1×10^6^ cells/ml), spun down at 1500 g for 10 minutes, resuspended in 20 µl IMDM medium and subsequently added to 100 µl of *Renilla* Luciferase Assay (1 µl of 100× *Renilla* Luciferase Assay substrate dissolved into 100 µl of *Renilla* Luciferase Assay Buffer). The level of *Renilla* luciferase activity (RLU) from 1×10^6^ samples was monitored at different time points after substrate addition in a luminometer. To monitor the signal of lysed cells, the same amount of cells was lysed and measured in the same system according to manufacturers instructions.

### *In vivo* bioluminescence imaging

Mice were anaesthetized with 2.3% isoflurane. At different days after infection, mice were injected intraperitoneally with 100 µL of coelenterazine (2 µg/µl dissolved in methanol) (Synchem) diluted with 90 µL PBS pH 7, anaesthetized with isoflurane and light emission in photons/second/cm^2^/steradian (p/sec/cm^2^/sr) was recorded in an IVIS Imaging System 100 (Xenogen LifeSciences) and Living Image® 2.20.1 software (Xenogen) for 180 seconds. Measurements started 3–5 minutes after substrate injection to allow the spread of the coelenterazine.

### Immunohistochemistry

Mice at 25 days after infection were deeply anaesthetized with isoflurane, sacrificed and testes were dissected. To examine presence of trypanosomes within and outside blood vessels in the testis, sections were cut, mounted, fixed and immunostained with anti-AnTat 1.1 VSG (1∶5.000) and goat polyclonal anti-glucose transporter 1 (1∶40; GLUT-1, Santa Cruz Biotechnology, Santa Cruz, CA, USA) as described previously [11]. Glut 1 usually used to stain cerebral blood vessels has also been shown to be expressed by testicular endothelial cells [12]. Sections were examined and analysed using a Nikon fluorescence microscope. Photomicrographs were taken with a Zeiss AxioCam digital camera.

### Parasite viability by oxidation to formazan

WST-1(tetrazolium salt) salts are cleaved to formazan by cellular oxidoreductases. The augmentation in enzyme activity lead to an increase in the amount of formazan dye formed. The viable cells were quantified by the formazan dye produced by metabolically active cells. To measure the drug sensitivity of cordycepin and other drugs, 25000 parasites were cultured in 100 µl *in vitro* in a 96 well culture plate with serial drug dilutions. Viability of the parasites was measured by adding 10 µl of WST reagent and further incubation for 2 hours. Readings were taken by a multiwell scanning spectrophotometer at excitation wavelength of 450 nm.

### CFSE assay

In order to measure single cell proliferation trypanosomes at different days of culture CFSE labeling was performed as described for mammalian cells [13]. To each milliliter of cell suspension, 2 µl of carboxyfluorescein diacetate, succinimidyl ester or CFDA-SE (CFSE) 5 mM stock solution was added and immediately mixed to ensure uniform staining, resulting in a final concentration of 10 µM CFSE. The cells were incubated 15 min at 37 C and the cells were quenched by adding 5 volumes of culture medium. Cells were analysed by flow cytometry with logarithmic detection of green fluorescence.

### Animal welfare and ethics

All mice were housed in filter-top cages and maintained in SPF barrier facilities in individual ventilated cages at the Karolinska Institute, Stockholm, or at the Institute for Tropical Medicine Antwerp. Animal ethics approval for the infection of live animals with recombinant trypanosomes was obtained from the respective Animal Ethical Committees of the Karolinska Institute (Sweden) and the Institute of Tropical Medicine, Antwerp (Belgium).

## Results

### Construction of luminescent *T. brucei*, expression and localisation of *Renilla* luciferase in the parasite

For stable transfection of *Renilla luciferase* (Rluc) into the β-tubulin region of the bloodstream form of monomorphic *T b. brucei* Lister 427 and pleomorphic *T. b. brucei* AnTat 1.1, the Rluc gene was PCR-amplified and cloned into the pHD309 plasmid [8]. Plasmids were screened via HindIII/BamHI double restriction-digestion, sequenced, and those with the correct insert in frame were selected and propagated in *E. coli*. For transfection, 2×10^7^ parasites were electroporated with NotI linearized DNA construct in a BioRad Gene Pulse Xcell square wave electroporator. Two independent transfections were performed and three clones from each population were selected for further luminescence experiments

### *In vitro* bioluminescence, uptake of substrate and light emission of live cells

**Figure 1:**
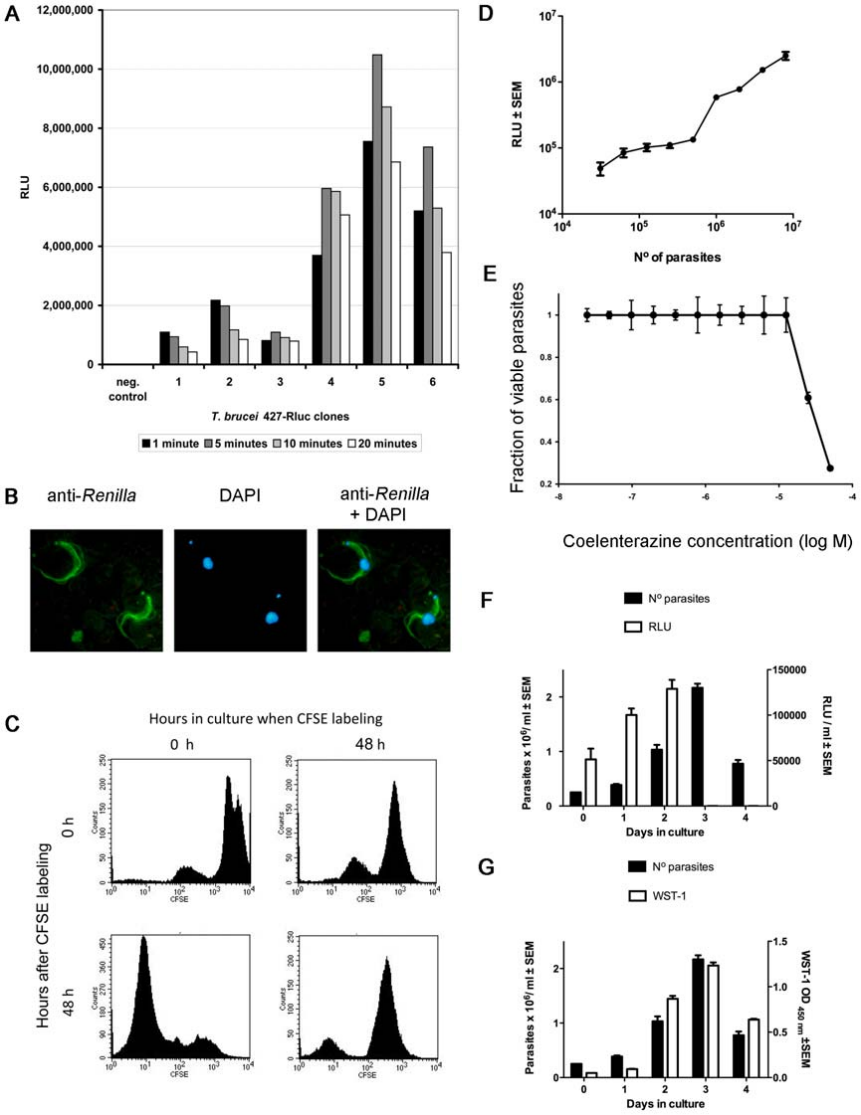
*In vitro* bioluminescence, uptake of substrate and light emission of live cells. A. *In vitro* activity of 6 clones of *T.b. brucei* 427-Rluc expressing the *Renilla luciferase* is shown at different time points after substrate addition. Clones 1–3 and 4–6 were selected from two independent electroporation events. Efficiency of *Renilla luciferase* expression in clones 1–3 is lower than clones 4–6, yet resulting in a detectable signal. Clone 4 was selected for further *in vivo* work, showing a high RLU signal slowly decreasing over time. Negative control corresponds to non-transformed *T.b. brucei* 427. B. Correlation between parasite concentration and RLU activity. Different concentrations of Rluc transfected *T.b. brucei* Lister 427 were incubated with coelenterazine and RLU of triplicate wells measured after 5 minutes incubation. C. Expression and localization of *Renilla* luciferase in *T. brucei* AnTat 1.1E. Anti-Renilla FITC staining (left), DAPI staining (middle) and overlay (right). Magnification: 60×. D. *T.b. brucei* Lister 427 was incubated for 72 h with different concentrations of coelenterazine and the viability of triplicate cultures of the parasites was determined by WST-1 assay in relation to untreated parasites. The arrow indicates the final concentration of coelenterazine used *in vivo*. E. Luciferase activity in different life stages of *T. b. brucei* AnTat1.1^E^. Parasites were labeled with CFSE at 0 or 48 h after initiation of the culture. At 0 or 48 h after labeling cells parasites were fixed and CFSE dilution in daughter cells was analysed by FACS. F, G. *T. b. brucei* AnTat1.1^E^ density, WST-1 assay and RLU were measured in triplicate at the indicated time points culture of (2.5×10^4^) parasites.

The kinetics of luciferase activity of *T. brucei* Lister 427 and AnTat 1∶1 clones showed a fast reaction and prolonged response during time. Both live and lysed cells showed a high relative luciferase units (RLU) activity, although the RLU signals for lysed cells were about 5 to 10 times higher (Figure 1A and data not shown). A linear relationship between concentration of live parasites and the RLU could be observed (Figure 1B). The *Renilla* Luciferase Assay System (Promega) was used to measure *in vitro* luciferase activity of live and lysed parasites.

Subsequently, we verified whether the RLU signal measured from live cells was due to substrate uptake and not residual activity of free luciferase released from damaged or live cells during manipulation. First, cells were spun down, the supernatants collected and the cell pellet resuspended and luciferase activity measured in supernatants and cell pellets. Over 70% of the original light emission was generated by the cell pellet whereas a negligible signal was detected in the supernatant (data not shown). As a second control, FACS analysis performed on 10^6^ non-lysed parasites incubated with propidium iodide, a marker for non-viable cells, showed no incorporation of the dye, indicating that over 99% of the cells were intact after manipulations (data not shown). Taken together, these results suggest that the luciferase substrate coelenterazine penetrates or is taken up by live trypanosomes and that the detected luciferase is not secreted or released by the trypanosomes.

*Renilla* luciferase was expected to locate in the cytoplasm. Indeed, luciferase was immunostained throughout the whole cell with a slightly higher concentration around the flagellum (Figure 1C).

We then investigated if coelenterazine is toxic for parasites since this could hamper the follow up of the infection *in vivo*. Parasites were grown *in vitro* in the presence of different concentrations of coelenterazine and parasite growth was measured during 72 hours. Coelerenterazine at concentrations required for *in vivo* usage did not inhibit parasite proliferation (Figure 1D).

*T. brucei* undergoes a life cycle stage differentiation from a long slender to a short stumpy form [14]). We analysed whether slender and stumpy forms of *T. brucei* express luciferase activity and cleave WST-1. Parasites were alive in our culture condition until day 4–5 of culture. Exponential growth of parasites was observed during the first 48 h in cultures whereas at day 3–4 similar parasite concentrations were observed. Confirming previous data [14], the lack of increase in parasite density in the cultures was due to an arrest in proliferation rather than to an increased parasite death, as visualized by the dilution of CFSE by parasites during the days 1 and 2 but not 3 and 4 of culture (Figure 1 E). Long slender had lower WST-1 cleavage ability per cell than stumpy forms (Figure 1 G), whereas stumpy forms showed negligible luciferase activity compared to long slender forms (Figure 1 F).

### *In vivo* bioluminescence, model validation and tropisms observed

**Figure 2:**
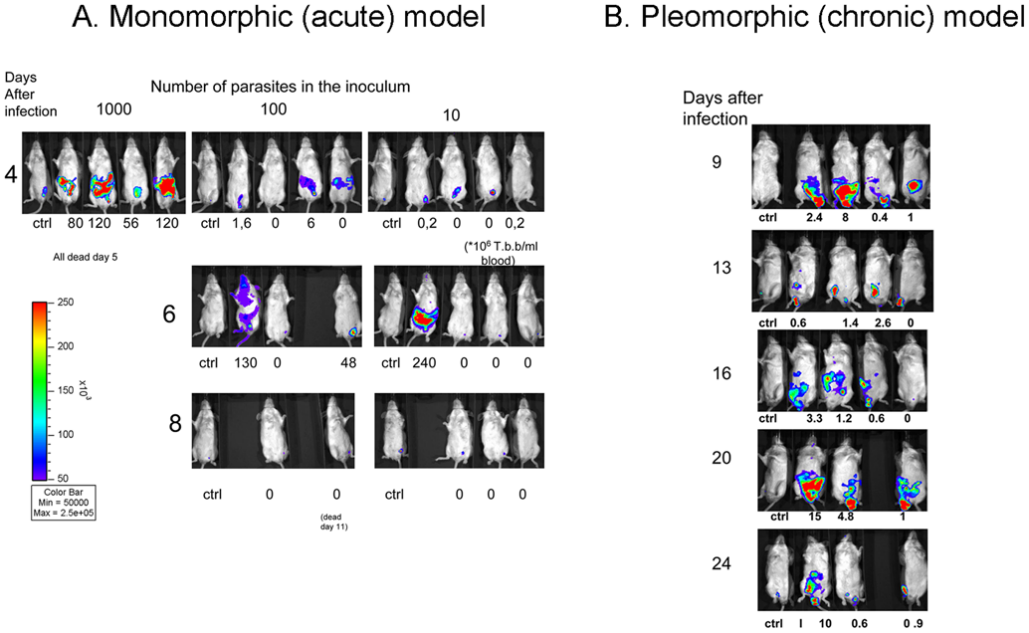
*In vivo* bioluminescence models. A. Dynamics of *T.b. brucei* Lister 427 infection in female BALB/c mice. Balb/c mice were infected i.p. with the indicated numbers of *T.b. brucei* lister 427. Coelenterazine was inoculated 3–5 minutes before light detection in an IVIS Imaging System. This procedure was repeated at different days after infection. A non infected mouse is included as a control on the left of each image set. Color scales indicate photon emission (photons s−1 cm−2) during a 180-s exposure. The parasitemia levels of individual mice at the specific time points are also indicated (×10^6^/ml). B. Dynamics of *T.b. brucei* AnTat 1.1 infection visualized by bioluminescence imaging. Parasite load in BALB/c male mice injected i.p. with 2×10^4^ luciferase recombinant *T.b. brucei* AnTat 1.1 was assessed daily by biophotonic emission determination as indicated in material and methods. Parasitemia levels were registered in parallel (×10^6^/ml).

The outcome of infection with the monomorphic *T. brucei* Lister 427 in female mice was then studied. Mice infected i.p. with 10, 100 or 1000 parasites were inoculated i.p. with 20 µg/kg coelenterazine 2–4 minutes before light measurement. All mice died 5 days after infection with 1000 *T. b. brucei*, while when inoculated with 10 or 100 parasites mice survived up to day 11 after infection (Figure 2A). A fraction of mice survived that inoculation. With exception of one animal, none of the surviving animals showed detectable parasitemia. Light emission, usually located in the peritoneal cavity, was observed in all mice showing positive parasitemia (Figure 2A).

**Figure 3:**
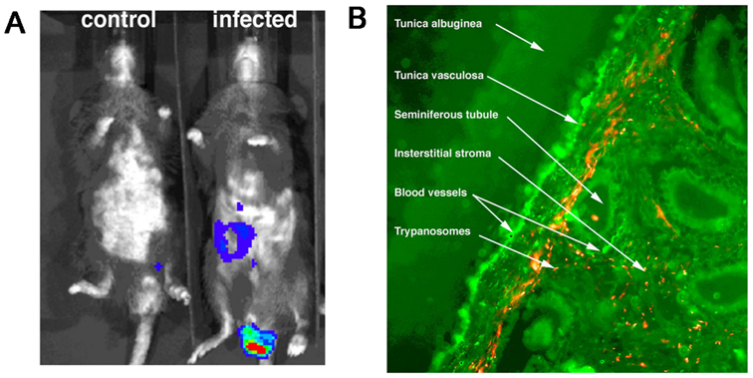
Testis localization of bioluminescent *T. b. brucei*. A. RAG1^−/−^ mice at 0 (left) and 7 days after infection (right) with 2×10 ^4^ *T. brucei* Lister 427 were inoculated with coelenterazine and biophotonic emission was assessed. B. Immunostaining of trypanosomes and endothelial cells in sections of testis from mice 25 days after infection with 2×10^4^ *T.b. brucei* Antat 1.1E i.p. Numerous parasites (in red) are observed in the blood vessels and in the interstitial tissue between the seminiferous tubules. Magnification: 250×.

The inoculation of BALB/c male mice with 2.10^4^ pleomorphic luciferase tagged *T. b. brucei* AnTat 1.1E resulted in a prolonged survival, similar to that observed after infection with the isogenic non-recombinant parasites (data not shown). Mice showed signs of morbidity circa 3 weeks after infection but no increased light emission or parasitemia. The intensity of light emission was not always associated with parasitemia levels. Interestingly, a preferential localization of parasites in the testis was detected in several animals infected with *T. b. brucei* AnTat 1.1^E^ (Figure 2B), an observation that was reproduced when infecting male BALB/c mice with *T. brucei* Lister 427 (data not shown). When performing bioluminescence experiments in female mice, no apparent sequestration to the sexual organs (*in casu* the ovaries) was observed (data not shown). We studied if the pressure exerted by the adaptive immune responses determined preferential localization in the testis. For this purpose we infected B- and T cell-deficient RAG1^−/−^ mice with 100 *T. b. brucei* Lister 427 recombinant parasites. RAG1^−/−^ mice also showed testis localization of *T. b. brucei* after infection indicating that testis localization is probably due to parasite tropism for testis or enhanced parasite growth in this organ (Figure 3A).

The immunostaining of trypanosomes in the testis of mice 25 days after infection with *T. b. brucei* AnTat 1.1^E^ confirmed the information provided by the bioluminescent technique. Trypanosomes were observed within and outside blood vessels, in the interstitial stroma between seminiferous tubules (Figure 3B).

**Figure 4:**
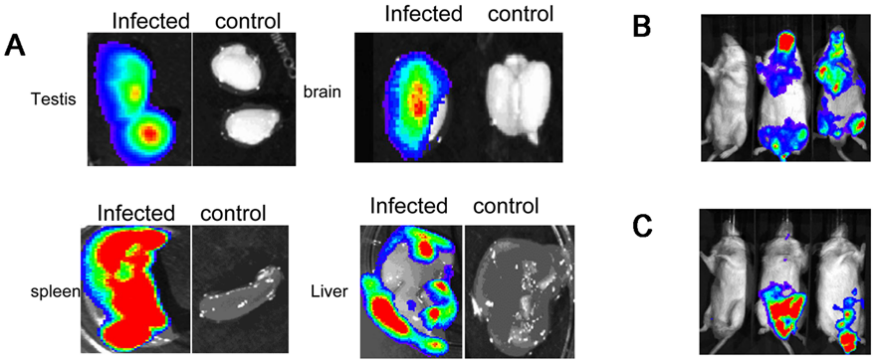
The light production in organs and after intraperitoneal (ip) and intravenous (iv) substrate inoculation. A. Imaging of bioluminescent parasites in organs from BALB/c mice infected i.p. with 2×10^4^ *T.b. brucei* AnTat 1.1 and uninfected controls 20 days after infection. Coelenterazine was added to all organs before light detection *ex vivo*. B, C. Real time in vivo imaging of light producing *T.b. brucei* AnTat 1.1 in mice at day 20 after infection. Coelenterazine was inoculated i.v. (B) or i.p. (C) 2–4 minutes before light detection.

In the experiments described above, biophotonic emission could be mainly detected in the abdominal cavity, and less frequently in the thorax and head of infected animals. Whether such localization was due to a preferential dissemination of the parasite in the abdomen and pelvis or to a non-homogenous distribution of coelenterazine *in vivo* was investigated. To analyze these possibilities mice infected with *T. b. brucei* AnTat 1.1^E^ were sacrificed and light production measured in organs after incubation with the substrate *ex-vivo*. Light was detected in the brain, spleen, lung and testis and to a lesser extent in the liver of infected mice. No light emission was detected in uninfected control animals (Figure 4A). Thus, a non-homogeneous distribution of coelenterazine after inoculation probably accounted for the light production pattern observed *in vivo*. Hence, we compared the light production after intraperitoneal (ip) and intravenous (iv) inoculation of coelenterazine into mice infected with *T. b. brucei* AnTat 1.1^E^. While an abdominal localization of light emission was detected in mice inoculated i.p. (Figure 4B) with coelenterazine, the iv inoculation of the substrate resulted in a thorax and cranial localization, suggesting an incomplete body distribution of the substrate by either route (Figure 4C).

### Application of the bioluminescent model: *In vitro* and *in vivo* drug efficacy testing

**Figure 5:**
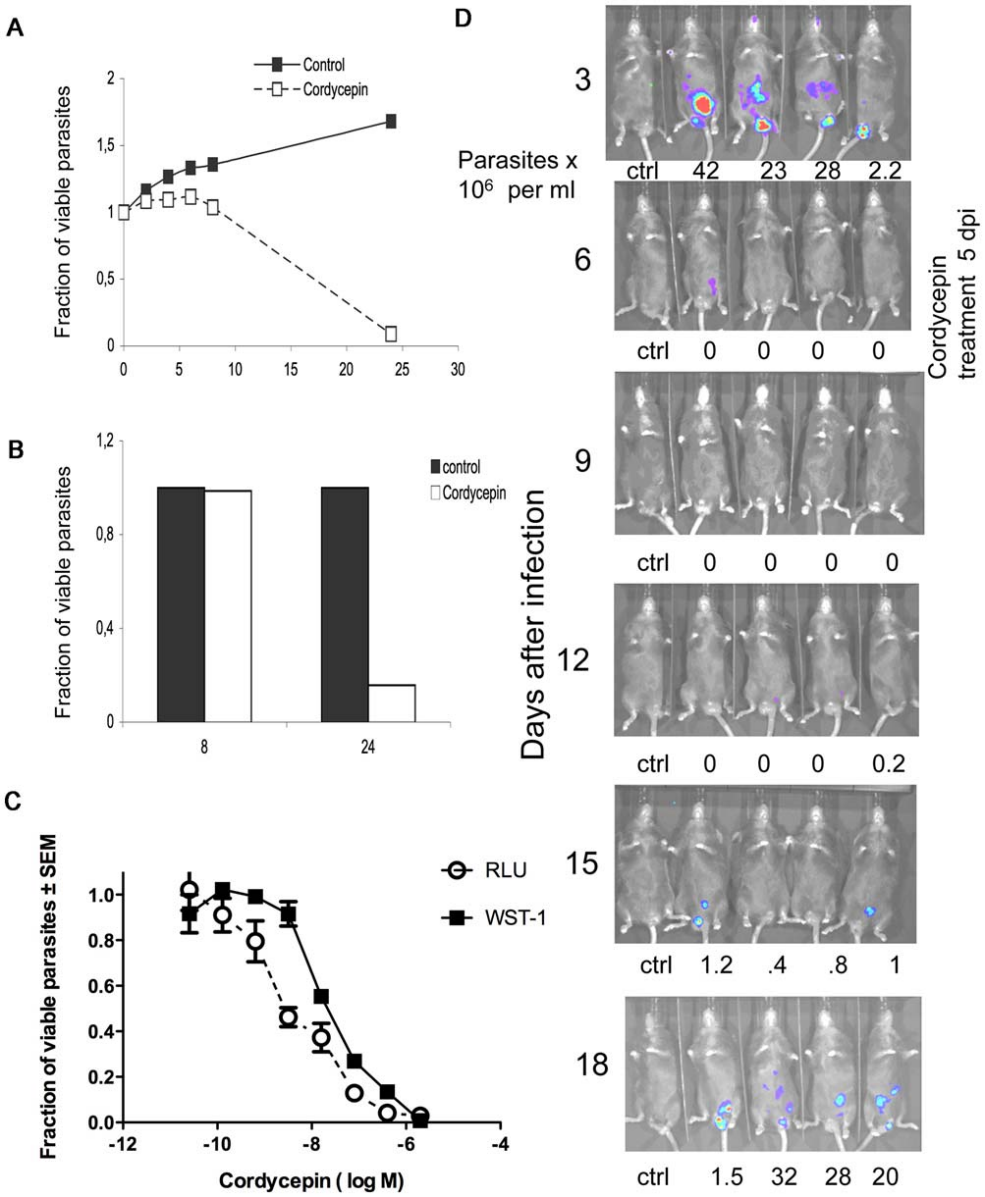
A sub-curative treatment with cordycepin and deoxycoformycin shows light emission in the testis. A, B. Parasites (10^6^ per ml) were incubated with 2 µM cordycepin or medium. The light production (A) or the viability as determined by a WST-1 assay (B) measured in triplicate cultures at different time points (in hours) after incubation. Results are expressed as fraction of the controls determined at 0 h of incubation. C. *T.b. brucei* Lister 427 (2×10^4^ cells) were incubated with different concentrations of cordycepin. The RLU and the parasite viability were determined 72 h after incubation. Results are expressed as fraction of each parameter in relation to the untreated controls. D. Kinetics of infection of RAG1^−/−^ male mice with recombinant *T.b. brucei* Lister 427. RAG1^−/−^ mice were infected ip with 100 *T.b. brucei* Lister 427. Five days after infection mice were treated i.p. with 2 mg/kg cordycepin daily for 3 days. Parasite load was measured by bioluminescence imaging and parasitemia was registered.

Whether recombinant parasites could be used for testing the efficiency of trypanocidal compounds *in vitro* was then studied. Light detection and parasite viability at different time points after incubation with the trypanocidal adenosine analogue cordycepin showed similar kinetics (Figure 5A, B). Parasites were also incubated with different concentrations of cordycepin and both, luciferase activity and parasite viability were equally diminished at similar concentrations of cordycepin (Figure 5C).

**Figure 6:**
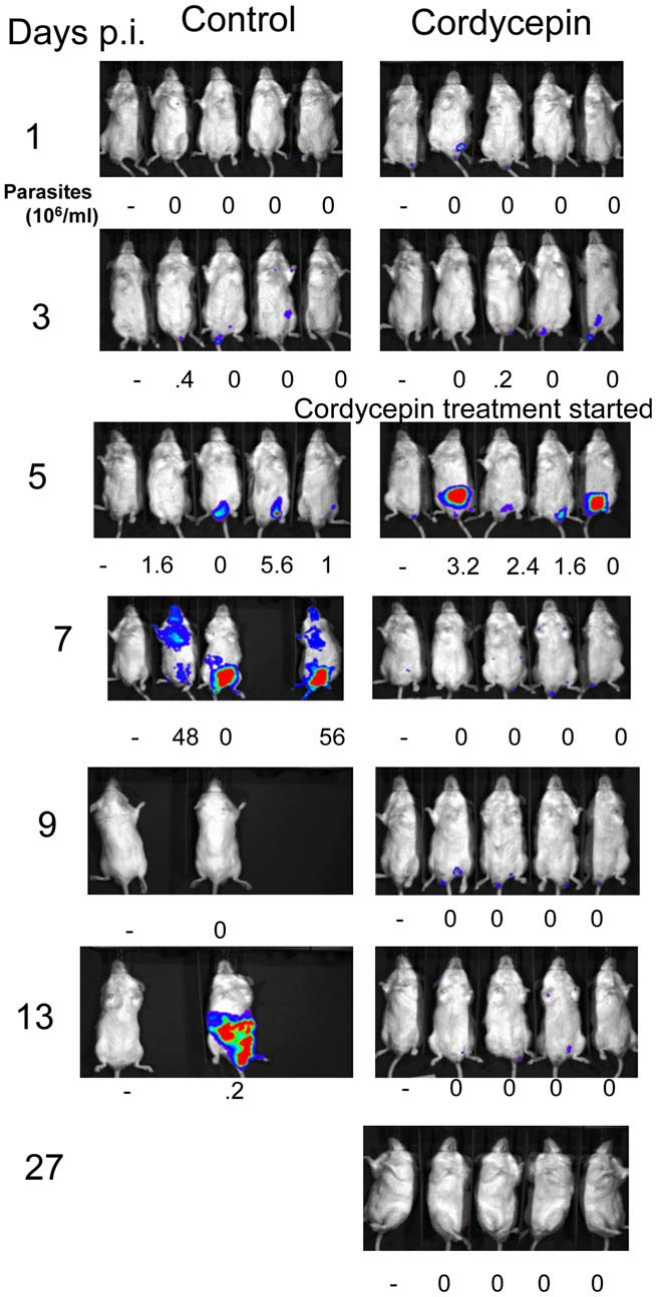
Curative treatments with cordycepin and deoxycoformycin shows disappearing light signals. Kinetics of infection of BALB/c male mice with recombinant *T.b. brucei* Lister 427. BALB/c mice were infected ip with 100 *T.b. brucei* Lister 427. Five days after infection mice were treated i.p. with 2 mg/kg cordycepin daily for 7 days. Parasite load was measured by bioluminescence imaging and parasitemia was registered.

Subsequently, the luciferase-labeled parasites were used to validate the biophotonic method for testing of trypanocidal compounds *in vivo*. Treatment with 7 doses of cordycepin and the adenosine deaminase inhibitor deoxycoformycin cures experimental infections with *T. b. brucei* [15]. A sub-curative treatment with cordycepin and deoxycoformycin in RAG1^−/−^ mice infected with luciferase tagged strains resulted in waning of biophotonic emission (Figure 5D). Several days after treatment, light production and parasitemia were detectable. Some mice showed light production in testis suggesting that *T. brucei* are protected by the testis-blood barrier from suboptimal doses of trypanocidal drugs (Figure 5D). In contrast, neither parasitemia or light emission were detected in luciferase-tagged *T. b. brucei* infected BALB/c mice treated daily for 7 days with cordycepin and deoxycoformycin starting 5 days after infection (Figure 6).

## Discussion

In this paper, we demonstrate for the first time the feasibility of detecting live trypanosomes through real-time *in vivo* and *ex vivo* luminescence imaging. We opted to use *Renilla* luciferase rather than firefly luciferase since previous studies in procyclic trypanosomes (insect stage) showed that the firefly luciferase accumulated in glycosomes [16]. This may impede the growth of bloodstream mammalian forms due to major changes in the energy metabolism and thereby hamper *in vivo* bioluminescence studies (George Cross, personal communication). There may be two reasons why the *Renilla* luciferase worked so well *in vivo* (i) the substrate coelenterazine is less polar than d-luciferin, and might pass through the cell membranes more readily and (ii) the C-terminus of *Renilla* luciferase (VLKNEQ) does not appear to have a peroxisomal targeting sequence, whereas firefly has the classic GGKSKL. Hence, we showed that *Renilla* luciferase was located in the cytoplasm where the substrate accumulates and does not hamper energy metabolism in the glycosome. A dose of 20 µg coelenterazine was used as a substrate, as described previously for the monitoring of metastasis in mice using bioluminescence [17]. Of importance is the stage-dependent luciferase activity, being significantly lower in stumpy than in slender forms. The transition of slender into stumpy bloodstream forms includes cell cycle arrest and a decrease in protein synthesis, probably due to decrease polysome formation [18] that could account for lower luciferase activity in this life stage. On the contrary, the stumpy forms showed increased WST-1 reduction probably attributed to the increased levels of oxidoreductases in this form compared to long slender forms [19].

Analogous to other models [20], live *T. b. brucei* produced light after addition of substrate and distinct temporal differences in light production were revealed following intravenous or intraperitoneal delivery. Bhaumik and Gambhir [21] stated in their discussion that biodistribution of coelenterazine and the potential toxicity of repetitively using coelenterazine in living mice should be further investigated. They hypothesized that is was likely that coelenterazine will be accessible to many tissues because of its diffusible nature. We found that repeated injection of coelenterazine did not show toxic effects on the mice. However, the distribution of coelenterazine *in vivo* seemed not homogenous and depended largely on the way of administering the substrate. This is in accordance with recent findings [22] which showed that intranasal administration of luciferin rather than intraperitoneal injection increased the sensitivity of detecting nasal and pulmonary airway infections by a 30-fold. Hence the route of substrate administration should be considered in the interpretation of the real time images. According to the tissues of interest, either intraperitoneal, intravenous, or a dual injection, should be considered. Another possibility would be to increase the dose of substrate to verify if the local tissue concentration of coelenterazine is sufficient to give a detectable signal. According to the toxicity assays (Figure 1D) it would be possible to increase the dose by a 10-fold.

The lack of toxicity of coelenterazine for parasites and the host at the doses used *in vivo*, and the lack of light emission by killed recombinant parasites support the strength of luciferase-tagged parasites to study *in vivo* parasite dissemination as well as drug compound screening, both *in vitro* and *in vivo*. The Rluc-pHD309 plasmid integrates at the conserved β-tubulin of the *Trypanosoma* species, hence other *T. brucei* strains and taxa can easily be transfected with the *Renilla* luciferase marker resulting in new models to monitor drug sensitivity and the spread of parasites in a murine model.

A very interesting finding was the abundance of parasites in the testis. *T. b. brucei* parasites could be observed extravascularly in testis but not in the seminiferous duct, suggesting sexual transmission is unlikely. Accordingly, we observed that no female immunodeficient mice became infected when mated with *T. brucei*- infected BALB/c mice. Of interest, the natural transmission of *Trypanosoma equiperdum* closely related to *T. brucei*, occurs during copulation [23]. The distribution of trypanosomes in the testicular tissue is in accordance with a previous study showing that trypanosomes were present in the intertubular tissues, but never crossed the basal lamina of seminiferous tubules [24]. In that study, necrosis of cells in the seminiferous tubule and a mononuclear infiltration in the interstitium was noted. It might be that in *T. equiperdum* infections in equines this may contribute to disease transmission. Biophotonic real time detection of parasite dissemination will be useful to study *T. equiperdum* models to examine tissue tropisms and transmission routes.

We should note that the current model uses intraperitoneal injection which may somehow bias the observed dissemination of the parasites. In future models it may be interesting to perform subcutaneous infections which mimic tsetse delivery.

The possibility that parasites have a preferential tropism for testes can also be of importance when considering drug development, since parasites might be protected from many drugs by the blood-testis barrier. In line with this, parasites were detected in testes upon reactivation of the infection in mice treated with sub-curative doses of cordycepin and deoxycoformycin.

It could be further speculated that the proximity of parasites to Leydig cells located in the interstitial tissues might affect the endocrine balance, contributing both to the pathology of disease. In line with this idea, testosterone levels, testicular responsiveness to exogenous gonadotropin and number of testicular LH receptors were reduced in *T. b. brucei* infected rats indicating gonadal imbalance [25]. Decreased concentrations of testosterone were detected in patients with human African trypanosomiasis [26]. In accordance, we observed that no offspring was generated after mating 4 male mice 20 days after infection with *T. brucei* AnTat1.1E with 2 uninfected females each for 10 days. All females remained uninfected, suggesting male sterility and absence of sexual transmission of the parasites. The preference of parasites for the testes does not appear to be a result of immune pressure since it also occurred in RAG1^−/−^ mice, lacking B and T cells.

In conclusion, this bioluminescent model opens new avenues to examine the dissemination of parasites of different *Trypanosoma* species into different organs, and the *in vivo* monitoring of drug efficiency.

## Supporting Information

Alternative Language Abstract S1 Translation of the Abstract into French by Philippe Büscher
(0.03 MB DOC)

Alternative Language Abstract S2 Translation of the Abstract into Spanish by Martin Rottenberg
(0.03 MB DOC)
