# Supplementary material for: Bioluminescent Imaging of Trypanosoma brucei Shows Preferential Testis Dissemination Which May Hamper Drug Efficacy in Sleeping Sickness
Source: PLoS Negl Trop Dis. 2009 Jul 21;3(7):e486. doi: 10.1371/journal.pntd.0000486 (PMC2707598; doi:10.1371/journal.pntd.0000486)
Supplement: Alternative Language Abstract S1 — Translation of the Abstract into French by Philippe Büscher (0.03 MB DOC) [file pntd.0000486.s001.doc]

**résumé**

L'imagerie *in vivo* en temps réel est une méthode rapide pour suivre la dissémination de *Trypanosoma* dans leur hôte, particulièrement dans les organes riches en tissus lymphoïdes. Dans ce manuscrit, nous rendons compte des souches récombinantes monomorphes et pléomorphes de *Trypanosoma bruce*i exprimant la luciférase de *Renilla*.

Les expériences *in vitro* confirment l'internalisation du substrat coelenterazine par les parasites vivants ainsi que l'activité de la luciférase. En plus, nous montrons qu'il est possible, par imagerie *in vivo*, de suivre dans des souris infectées, les trypanosomes rendus bioluminescents par la luciférase de *Renilla.*

Il est intéressant de noter le tropisme préférentiel de ces 2 souches pour les testicules des souris. Ce phénomène est important pour le développement de nouveaux médicaments étant donné que les parasites pourraient ne pas être accessibles aux médicaments du fait de la barrière hémo-testiculaire. Cette hypothèse est corroborée par les expériences sur l'efficacité des trypanocides dans des souris infectées avec le *Trypanosoma brucei*. En effet, il a été demontré que les parasites sont éliminés moins vite dans les testicules que dans la cavité abdominale.
