# Supplementary material for: Bioluminescent Imaging of Trypanosoma brucei Shows Preferential Testis Dissemination Which May Hamper Drug Efficacy in Sleeping Sickness
Source: PLoS Negl Trop Dis. 2009 Jul 21;3(7):e486. doi: 10.1371/journal.pntd.0000486 (PMC2707598; doi:10.1371/journal.pntd.0000486)
Supplement: Alternative Language Abstract S2 — Translation of the Abstract into Spanish by Martin Rottenberg (0.03 MB DOC) [file pntd.0000486.s002.doc]

**Author summary, Spanish translation**

La Tripanosomiasis Humana Africana o enfermedad del sueño, infección causada por dos subespecies de Tripanosoma Brucei, es endémica en el África Subsahariano. Contra estas infecciones no existe vacunación. Por otra parte, las drogas poseen importantes efectos secundarios, que en algunos casos son letales. En la actualidad, se desconoce cómo y cuándo el parásito abandona los vasos sanguíneos y penetra diferentes órganos (en especial el cerebro). Este conocimiento puede contribuir a desarrollar y verificar nuevos compuestos que puedan eliminar al parásito de la sangre y los tejidos.

En este estudio, describimos el desarrollo de una nueva técnica que permite medir la presencia de parásitos en tiempo real y en vida del huésped infectado, a través de la detección de señales luminosas emitidas por aquellos. Utilizando este método demostramos que en un estadio temprano de la infección los parásitos migran a los testículos, sitio al cual no acceden la mayoría de las drogas parasiticidas ni los constituyentes humorales y celulares de la respuesta inmune. Estos parásitos podrán con posterioridad re-invadir otros órganos del animal infectado.
